# Considering Electrospun Nanofibers as a Filler Network in Electrospun Nanofiber-Reinforced Composites to Predict the Tensile Strength and Young’s Modulus of Nanocomposites: A Modeling Study

**DOI:** 10.3390/polym14245425

**Published:** 2022-12-11

**Authors:** Vishal Gavande, Saravanan Nagappan, Won-Ki Lee

**Affiliations:** 1 Division of Polymer Engineering, Pukyong National University, Busan 48513, Republic of Korea; 2 Department of Chemistry, Chemistry Institute for Functional Materials, Pusan National University, 2 Busandaehak-ro 63beon-gil, Busan 46241, Republic of Korea

**Keywords:** nanocomposites, electrospinning, nanofiber-reinforced composites, mechanical properties, modeling

## Abstract

In this study, a simple approach was described to investigate the theoretical models for electrospun polymer nanofiber-reinforced nanocomposites. For predicting the tensile strength of the electrospun nylon 6 nanofiber-reinforced polyurethane acrylate composites, conventional Pukanszky, Nicolais–Narkis, Halpin–Tsai, and Neilson models were used, while for Young’s modulus, Halpin–Tsai, modified Halpin–Tsai, and Hui–Shia models were used. As per the Pukanszky model, composite films showed better interaction since the values of the interaction parameter, *B*, were more than 3. Similarly, the value of an interfacial parameter, *K*, was less than 1.21 (*K* = −5, for the curve fitting) as per the Nicolais–Narkis model, which indicated better interfacial interaction. For composite films, the modified Halpin–Tsai model was revised again by introducing the orientation factor, *α*, which was 0.333 for the randomly oriented continuous nanofiber-reinforced composites, and the exponential shape factor, *ξ* = (2$l/d)e^{-av_{f}-b}$, which showed the best agreement with the experimental Young’s modulus results. Based on mentioned remarks, these models would be applicable for estimating the tensile strength and Young’s modulus of electrospun nanofiber-reinforced polymer composite films.

## 1. Introduction

Polymer nanocomposites reveal significant high-performance properties using small fractions of nanofillers in the polymer matrices. The excellent properties of nanocomposites have attracted extensive attention in distinct technologies such as automobiles, energy, sensors, fuel cells, agriculture, and biotechnology [1,2,3,4,5]. One of the attractive characteristics of polymer nanocomposites is their mechanical properties. However, the mechanical characteristics of composites cannot be predicted properly due to the small fractions and novel characteristics of the polymer nanocomposites, compared to traditional composites. The mechanical characteristics of polymer nanocomposites have been estimated with many proposed models [6,7,8].

During the last few decades, the electrospinning technique experienced substantial progress and attracted researchers from various fields, such as biomedical, sensors, energy, and environmental applications [9,10,11]. Many nanofiber fabrication techniques, such as drawing, template synthesis, temperature-induced phase separation, and molecular self-assembly, are not scalable, are limited to specific polymers, and are tricky to control the fiber dimensions for [12]. In the case of electrospinning, it offers distinct advantages, such as control over morphology, porosity, and ease of fiber functionalization and material combination; a wide variety of polymers and materials have been used to form nanofibers with a variety of nanofibrous structures involving simple equipment [9,13]. The excellent properties of electrospun nanofibers make them the perfect candidate for reinforcements in composite applications [14,15,16]. The electrospinning technique can consistently produce nano- to submicron-sized diameters with a high surface area to volume ratio, causing extended polymeric chain confirmations and highly crystalline regions of benign polymer structures with fiber mechanical characteristics. It provides more effective interfacial bonding with the matrix material.

Due to their higher molecular orientation and higher crystallinity, ultrafine-diameter nanofibers show improved mechanical performance, including tensile strength, Young’s modulus, and toughness compared to microfibers [17,18]. Many researchers have proved the size effect on the mechanical properties of the fibers. Chew et al. reported a dramatic increase in Young’s modulus from 300 MPa to 3.2 GPa, and a tensile strength from 20 MPa to 220 MPa, when the diameter of polycaprolactone (PCL) fibers decreased from 5 µm down to 200–330 nm [19]. Similarly, a reduction in fiber diameter from 2.8 µm to 100 nm resulted in a simultaneous increase in Young’s modulus from 360 MPa to 48 GPa, tensile strength from 15 MPa to 1.75 GPa, and toughness from 0.25 to 605 MPa, according to Papkov et al., when the diameter of the polyacrylonitrile (PAN) nanofiber was decreased down to the nanometer regime [20]. These effects could be explained by electric force-induced molecular orientation along the fiber axis during the electrospinning. A smaller diameter leads to higher crystallinity, and fewer surface defects per unit fiber length might appear on a smaller fiber, which results in better mechanical properties [15,21]. Due to their outstanding mechanical properties, polymer nanofibers are the best candidate for the reinforcement of polymers. Many researchers devoted their efforts to using electrospun polymeric nanofibers as reinforcement for the polymer matrices, in order to enhance their mechanical, thermal, and optical properties.

Recently, many electrospun nanofibers have been successfully employed to reinforce the polymer matrix. Many processes have been reported for the fabrication of nanofiber-reinforced composites, including solution impregnation, layer-by-layer coating, dip-coating, melt mixing with short electrospun nanofibers, in situ fabrication, casting, draining, and subsequent stacking by hot-press [9,15,22,23,24]. However, to fabricate electrospun nanofiber-reinforced composites, it is necessary to achieve an effective impregnation of the polymer matrix with the nanofibers, in order to establish intimate contact and strong interfacial bonding between the two, which then enables good mechanical interlocking with the surrounding polymer chains. Electrospun nanofiber-reinforced composites are widely used in human motion detection, self-healing electronic skins, sensors, fuel cells, epidermal electronics, microfluidic devices, biomedical applications, and so on. It would be beneficial to predict the mechanical properties of these composites with analytical and numerical modeling.

Polymer nanocomposites do not follow the rule of mixture for estimating mechanical properties, as conventional composites do. However, the mechanical characteristics of the composites cannot be estimated correctly due to the small fractions, novel size, and properties of the nanomaterials. Distinct models were suggested to predict the mechanical characteristics of nanofiller-reinforced polymer composites and short fiber-reinforced composites. However, not a single study (with either analytical or numerical models) has been published on the estimation of mechanical properties of nanofiber-reinforced composites. It is crucial to investigate the mechanical properties with modeling and simulation studies of the nanofiber-reinforced polymer nanocomposites. They became significant motifs because of the need for the advancement of these materials for in high-performance engineering applications. Gao et al. presented a numeric model to evaluate the elastic characteristics of the high-aspect-ratio short aramid fiber-reinforced composites [23]. Sun et al. reported on the mechanical characteristics of carbon nanofiber-reinforced epoxy resin composites and predicted the elastic modulus of the nanocomposites by employing the modified Halpin–Tsai equations [25]. Cox et al. reported a model and established an orientation factor for the randomness of the discontinuous fibers in the composite [26]. The notable properties of nanofiber-reinforced polymer nanocomposites were qualified to the robust interfacial adhesion between the polymer matrix and electrospun nanofibers, which properly transferred the load from the matrix to continuous nanofibers. The strong adhesion between the polymer and nanofibers from the interphase around the nanofibers was quite different from both matrix and nanofibers [24]. Our team has investigated the mechanical characteristics of the electrospun nylon 6 nanofiber-reinforced polyurethane acrylate nanocomposites [16]. In this regard, some wholesome models were proposed, which provide a possible means to determine mechanical properties in nanofiber-reinforced composites. The mechanical characteristics of the polymer nanocomposites depend on various parameters such as reinforcement, the aspect ratio of the nanofibers, the dispersion feature, and the filler volume fraction [27,28,29].

In this study, the ultimate tensile strength and Young’s modulus (*E_c_*) of the nylon 6 nanofiber-reinforced polyurethane acrylate nanocomposites (N6/PUA) were evaluated from experimental and theoretical views. The Pukanszky, Nicolais–Narkis, Halpin–Tsai, and Neilson models for the ultimate yield strength, and the Halpin–Tsai, modified Halpin–Tsai, and Hui−Shia model were used, assuming randomly oriented continuous nanofibers. Moreover, the assumptions of these models showed good agreement with the experimental results.

## 2. Experimental

### 2.1. Materials

A 15 wt% nylon 6 (1011 BRT, Hyosung, Seoul, Korea)/formic acid (Samchun Chemicals, Gyeonggi-do, Korea) polymer solution was used as a precursor solution to electrospun nanofibers using electrospinning units (NNC-ESP100, NanoNC Co. Ltd., Seoul, Korea). Difunctional polyurethane acrylate oligomer (Miramer PU2100, 7000 cPs at 25 °C) and isobornyl acrylate monomer diluent (Miramer M1140) were supplied by Miwon Specialty Chemicals Co. Ltd., Jeonbuk-do, Korea. Irgacure 184D (Ciba Specialty Chemicals, Basel, Switzerland) was used as a photocatalyst.

### 2.2. Method

The UV-curable polyurethane acrylate (PUA) matrix system was formulated with 50% difunctional polyurethane acrylate oligomer, 45% isobornyl acrylate monomer (as a diluent), and 5% of Irgacure 184D. Nylon 6 nanofiber-reinforced polyurethane acrylate nanocomposite films were fabricated by over-coating the nanofibers on the PUA matrix system, which consisted of casting, electrospinning, and UV curing. The formulated UV-curable PUA matrix system was cast on the glass substrate, and nylon 6 nanofibers were electrospun on the PUA-coated glass substrate for different deposition times (N15: 15 min, N30: 30 min, N60: 60 min, N2 h: 120 min, and N4 h: 240 min) without any break. Afterward, the nanocomposite was cured in a conveyor-belt-type UV-curing machine LZ-U101 (Make- Lichtzen Co. Ltd., Gunpo-si, Korea) fixed with a gallium lamp (160 w/cm, main wavelength: 365 nm, UV-A: 1100 mJ/cm^2^, Arc system). The detailed procedures of the electrospinning of nylon 6, fabrication of N6/PUA nanocomposites, and characterizations were reported in our earlier research [16]. The density of the nanofibers, PUA films, and N6/PUA nanocomposite films were calculated using ASTM D 792, with the help of a weighing balance using Archimedes principle.

## 3. Theoretical Models

There are three kinds of modeling concepts employed for polymer nanocomposites according to different size effects, such as molecular-scaled, micro-scaled, and meso-/macro-scaled models [6,30]. For the nanofiber-reinforced polymer composites, an applied force could be transferred from the polymer matrix to the polymeric nanofibers through shear stress at the nanofiber/polymer matrix interface.

### 3.1. Models for Tensile Strength

The Pukanszky model [28] explained the composition dependency of tensile strength in the nanocomposites, accepting the spontaneous formation of interphase as shown in the following:

(1) $$\sigma_{c}=\sigma_{m}\;\frac{1-\emptyset_{f}}{1+2.5\emptyset_{f}}\;exp\left(B\emptyset_{f}\right)$$

where *σ_c_* is the tensile strength of the composite, *σ_m_* is the tensile strength of the matrix, and $\emptyset_{f}$ is the volume fraction of polymer nanofibers. *B* is an interaction parameter denoting the load-bearing capacity of the filler, which depends on the interfacial interactions. The term $\frac{1-\emptyset_{f}}{1+2.5\emptyset_{f}}$ represents the effective load-bearing cross-section of the matrix. At zero interactions, the complete load is transferred by the matrix, and the load-bearing cross-section drops with an increase in the nanofiber content. If *B = 0*, the fillers act as voids, and due to this, the composite evidences inferior interfacial bonding, excepting adhesion and load transfer at the matrix–filler interface. However, if the value of *B ≤ 3*, the filler matrix interface is poor, apart from the reinforcing effect; from Equation (1), parameter *B* can be calculated with Equation (2):

(2) $$B=\frac{ln\;\left(\frac{\sigma_{c}}{\sigma_{m}}\;\frac{1+2.5\emptyset_{f}}{1-\emptyset_{f}}\right)}{\emptyset_{f}}\;$$

For the Nicolais–Narkis model, the ratio of the tensile yield stress of the composite (*σ_c_*) and tensile yield stress of a polymer matrix (*σ_m_*) deviates as a two-thirds power law function, with *K* as an interfacial parameter for filler matrix adhesion. Equation (3) is stated as follows:

(3) $$\frac{\sigma_{c}}{\sigma_{m}}=\left(1-K\emptyset_{f}{}^{\frac{2}{3}}\right)\;$$

The mechanical characteristics of the nanocomposites rely on the volume fraction (*ϕ_f_*), filler properties, structure, and interfacial interaction. If the adhesion between the filler and matrix polymer is not established, then the filler cannot bear the applied load, and the whole load is transferred to the matrix phase. In this equation, the parameter *K* counts for the adhesion between the reinforcement (filler) and matrix. The lower the *K* value, the stronger the adhesion. For an extreme case of weak adhesion, the theoretical value of *K* is 1.21 [27,29].

Conforming to this model, the tensile strength is stated, as in Equations (4) and (5), as follows:

(4) $$\sigma_{c}=\sigma_{m}\;\frac{1+A\eta \emptyset_{f}}{1-\eta \emptyset_{f}}$$

with,

(5) $$\eta =\frac{\frac{\sigma_{f}}{\sigma_{m}}-1}{\frac{\sigma_{f}}{\sigma_{m}}+A}$$

where *ϕ_f_* is the volume fraction of the nanofibers, and *σ_m_* and *σ_c_* are the tensile strength of the matrix and composite, respectively. Parameter *A* can be determined from the Einstein coefficient *K* [31], as stated below (Equations (6) and (7)):

(6) $$K=1+\frac{2l}{d}$$

with

(7) K=1+A

Similarly, the Neilson model is termed the modified Halpin–Tsai model, as Neilson revised the original equation by introducing *φ* as a filler packing factor. According to the Neilson model, the tensile strength is given as the following Equations (8) and (9):

(8) $$\sigma_{c}=\sigma_{m}\;\frac{1+A\eta \emptyset_{f}}{1-\eta \psi \emptyset_{f}}$$

where

(9) $$\psi =1+\left[\left(\frac{1-\varphi_{max}}{\varphi^{2}{}_{max}}\right)\right]\emptyset_{f}$$

*φ_max_* is the maximum packing fraction constant, and it is 0.82 for randomly oriented fibers.

### 3.2. Models for Young’s Modulus

The Halpin–Tsai Model anticipates the *E_c_* of several nanocomposites, where the reinforcement is in the form of nanofibers, nanotubes, nanorods, and nanoparticles with capricious aspect ratios [32,33,34,35]. The model can be illustrated as follows (Equations (10) and (11)):

(10) $$\frac{E_{c}}{E_{m}}=\frac{1+\tau \eta \emptyset_{f}}{1-\eta \emptyset_{f}}$$

with

(11) $$\eta =\frac{\frac{E_{f}}{E_{m}}-1}{\frac{E_{f}}{E_{m}}+\tau }$$

where *E_f_*, *E_c_*, and *E_m_* are the Young’s modulus of the fiber, composite, and matrix, respectively. *ϕ_f_* is the nanofiber volume fraction, and η is the shape factor relating to the reinforcement geometry. *τ* is the aspect ratio $\tau =2\left(l/d\right)$ for the tubular geometry. The length of the nanofiber was assumed to be *l* = 100,000 nm, and the diameter of the nanofiber was approximately 100 nm.

The Halpin–Tsai model (Equations (10) and (11)) correlates the modulus of the unidirectional fiber composites to the fiber volume fraction. This model cannot authentically predict the modulus of nanofiber-reinforced polymer nanocomposites, since it does not deliberate definite features of the nanofibers such as their high surface area, very high aspect ratio of nanofibers, dispersion of the nanofibers, and exceptional Young’s modulus of the nanofibers. However, nanofiber-reinforced polymer nanocomposites have a random alignment of the nanofibers in the matrix. Much research explored anticipating the modulus of randomly aligned fiber-reinforced composites by modifying the Halpin–Tsai model [33,36,37]. In some research, an orientation factor, *α*, accounted for the randomly oriented fiber. Likewise, in this case, the orientation factor, *α*, accounted for the randomly oriented fibers. If the length of the fiber is larger than the thickness of the samples, the fibers are assumed to be randomly oriented, and the value of the parameter is considered to be 0.333 (*α* = 1/3). If the length of the fiber is much smaller than the thickness of the specimen in randomly oriented fiber composites, then *α* = 1/6 is used [26,36,38,39]. However, the Halpin–Tsai model (Equations (12) and (13)) was modified as follows:

(12) $$\frac{E_{c}}{E_{m}}=\frac{1+\xi \eta \emptyset_{f}}{1-\eta \emptyset_{f}}$$

(13) $$\eta =\frac{\alpha \frac{E_{f}}{E_{m}}-1}{\alpha \frac{E_{f}}{E_{m}}+\xi }$$

Here, they further tailored the model by altering the shape factor, *τ*, to the *ξ = (2*$l/d)e^{-av_{f}-b}$, which is an exponential relation, with *a* and *b* being constants that are affiliated to the degree of fiber agglomeration, and by varying the values of *a* and *b*, the best fit to the experimentally determined *E_c_* [36,38].

The Hui–Shia model equation broadens the estimation of *E_c_* for unidirectional aligned composites with fiber- or flake-like fillers [31]. The Hui–Shia model eases the orientation of fibers, following the perfect interfacial bonding amongst matrix systems and nanofillers with similar Poisson’s ratios. The affiliated *E_c_* model Equations (14)–(20) are stated as follows:

(14) EcEm=E11Em=11−∅f4 1ξ’+3 ξ ’+Λ

(15) EcEm=E22Em=11−∅fξ’ 

with

(16)  ξ ’=∅f+EmEf−Em+31−∅f1−gα’2−g2α’2−1

(17) $$\Lambda =\left(1-\emptyset_{f}\right)\left[\frac{3\left(\alpha {’}^{2}-0.25\right)g-2{\alpha ’}^{2}}{{\alpha ’}^{2}-1}\right]$$

(18) $$g=\left(\frac{\alpha ’}{{\left(\alpha^{’}-1\right)}^{3/2}}\right)\left[\alpha^{’}{\left({\alpha^{’}}^{2}-1\right)}^{\frac{1}{2}}-cosh^{-1}\alpha ’\right]$$

(19) $$g=\left(\frac{\alpha ’}{{\left(1-\alpha^{’}\right)}^{3/2}}\right)\left[-\alpha^{’}{\left(1-{\alpha^{’}}^{2}\right)}^{\frac{1}{2}}+cos^{-1}\alpha ’\right]$$

(20) $$g=\left(\frac{\pi }{2}\right)\alpha ’$$

where *α’* is the converse of the aspect ratio (i.e., *d/l*), and *g* is assumed to be a geometric parameter of composites, which is expressed in Equations (18) and (19). Equation (18) refers to the incorporation of fiber-shaped fillers, while Equation (19) is applicable to the incorporation of flake- or plate-like nanofillers. Additionally, for a flawless interface, *g* would be stated in Equation (20).

## 4. Results and Discussion

**Figure 1:**
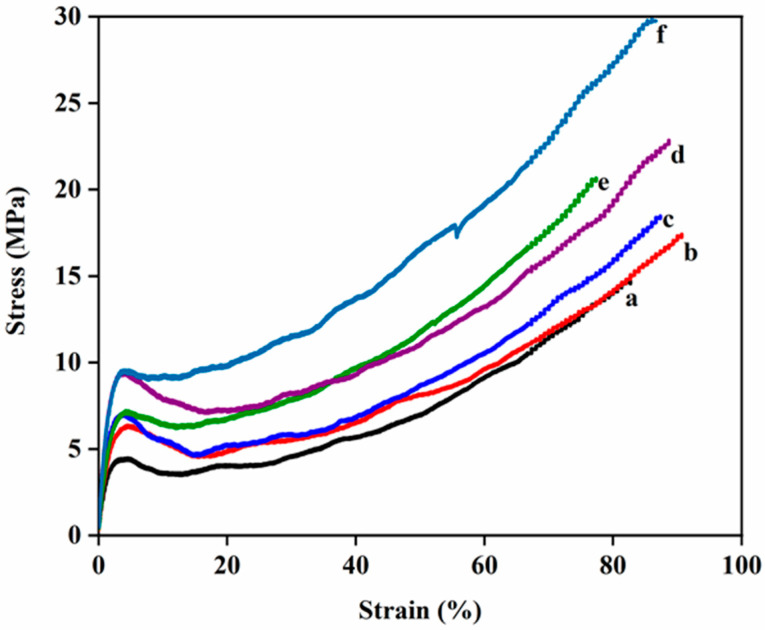
A typical example of the stress–strain curve of (**a**) reference PUA film, (**b**) N-15, (**c**) N-30, (publication-type="book") N-60, (**e**) N-2 h, and (**f**) N-4 h N6/PUA nanocomposite films (from ref. [16] copyright 2021 Wiley-VCH).

Table 1 shows the physical properties of the N6/PUA nanocomposites. Due to ultra-low loading (below 1%) of nylon 6 nanofibers in the PUA matrix, the practical and theoretical density of the nanocomposites did not show significant differences. Figure 1 shows the experimental stress–strain curves of the nylon 6 nanofiber-reinforced PUA nanocomposites. *E_c_*, tensile strengths, and % elongation were collected according to the average six sample estimates for an individual case. The mechanical characteristics of N6/PUA nanocomposites are noted in Table 2. The tensile strength and *E_c_* of the nanocomposites were enhanced with the introduction of nylon 6 nanofibers. There were no significant variations observed in % strains compared to the reference samples for each composite.

The discrepancy is much more familiar in modeling nanocomposite properties by the conventional rule of mixture or micromechanics models. In this section, we implemented the different models, and compared model predictions with the same set of available experimental data compiled in the previous study [16]. The material parameters required in the calculation process can be found in Table 1 and Table 2.

### 4.1. Tensile Strength of the Nanofiber-Reinforced Composites

The theoretical values of tensile strength were estimated with the Pukanszky model using Equations (1) and (2), and compared to the experimental values of nylon 6 nanofiber-reinforced PUA polymer nanocomposites. The constant *B* was determined by befitting experimental values with the mathematical values, and derived from the minimum sum of squares of variance more than from experimental values of composite strength. Pukanszky’s model underestimated the experimental data with the tensile strength of nanofiber-reinforced nanocomposites, when constant *B = 0*. It means that the nanofiber-reinforced nanocomposites dominated with better interfacial adhesion amongst the polymer nanofibers and matrices, and thus amelioration in tensile properties was depicted with the embodiment of reinforcement of the nanofibers. For the curve fitting, the constant *B* values were calculated by employing Equation (2), constant *B* values were 6.82, 5.75, 5.05, 4.27, and 4.04 for the N-15, N-30, N-60, N-2 h, and N-4 h N6/PUA nanocomposites, respectively, which indicated that nanofiber-reinforced nanocomposites at a nanofiber content from 0.004 to 0.066 vol%, revealed better interfacial bonding, resulting in more effective filler–matrix load transfer.

**Figure 2:**
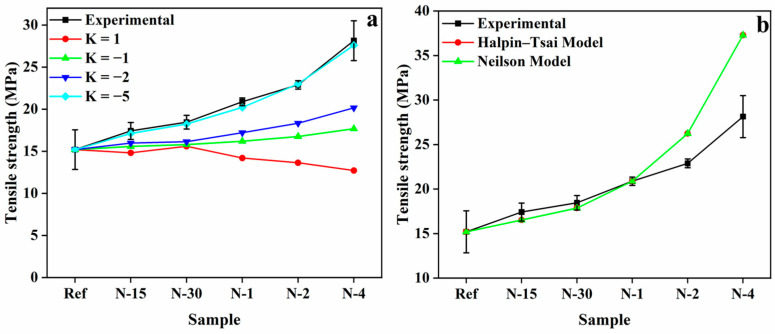
Correlations among the theoretical modeling values and experimental data of tensile strengths of nylon 6 nanofiber-reinforced PUA composites by (**a**) Nicolais–Narkis model, and (**b**) Halpin−Tsai model and Neilson model equations.

The theoretical predictions of tensile strength were compared employing the Nicolais–Narkis model Equation (3) and compared to the experimental values, as depicted in Figure 2. For modeling nylon 6/PUA matrix composites, the values of *K* were considered as 1, −1, −2, and −5 for the Nicolais–Narkis model. Using values of *K*, the tensile strength was estimated for the composites using Equation (1). As shown in Figure 2a, the experimental *K* values were approximately −5 for the N6/PUA matrix composites.

According to the Halpin–Tsai model, the tensile strength is predicted using Equations (4) and (5). However, the experimental and Halpin–Tsai model values showed higher bound values than the experimental values for the high-volume fraction of the nanofibers in composites. After modification of the Halpin–Tsai equation by Neilson with the introduction of factor *φ*, the values were quite similar to the Halpin–Tsai equation (Figure 2b).

### 4.2. Young’s Modulus of the Nanofiber-Reinforced Composites

**Figure 3:**
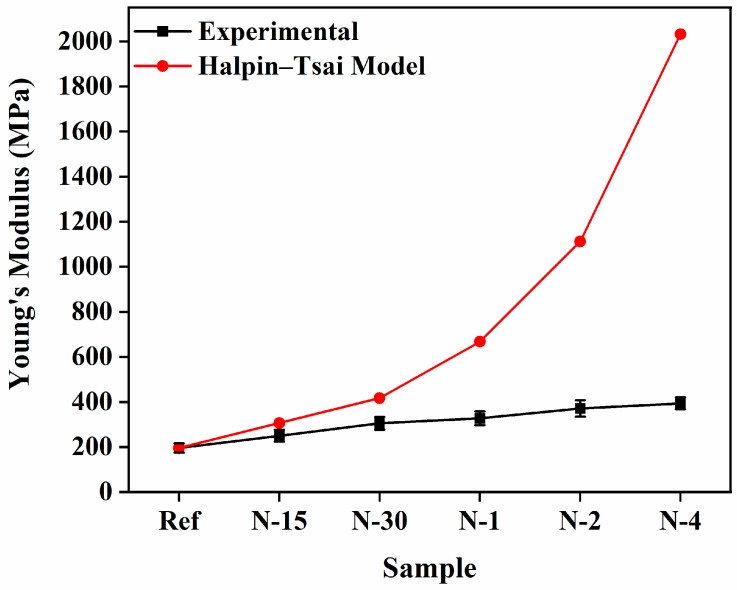
Comparison between the experimental *E_c_* and theoretical *E_c_* employing the Halpin–Tsai model.

The deviations of *E_c_* of the N6/PUA nanocomposites with the different volume fractions are plotted in Figure 3. To further determine the effectiveness of reinforcement, we implemented the conventional Halpin–Tsai model for N6/PUA nanocomposites. The Halpin–Tsai model is apparently effective in predicting *E_c_* of not merely unidirectional aligned fiber-reinforced composites, but also of several nanocomposites where the reinforcement phase has functionality relating to the aspect ratio in regards to CNF, CNTs, and cellulose nanofibers. As shown in Figure 3, the illustrated calculations show increments in *E_c_* as the volume fraction of nanofibers is enhanced. The experimental data of relative strength and the predictions of the model did not properly follow the experimental data at numerous nanofibers concentrations because of the absence of some parameters, such as an alignment of the nanofibers, shape factor, and aggregation in the composites.

**Figure 4:**
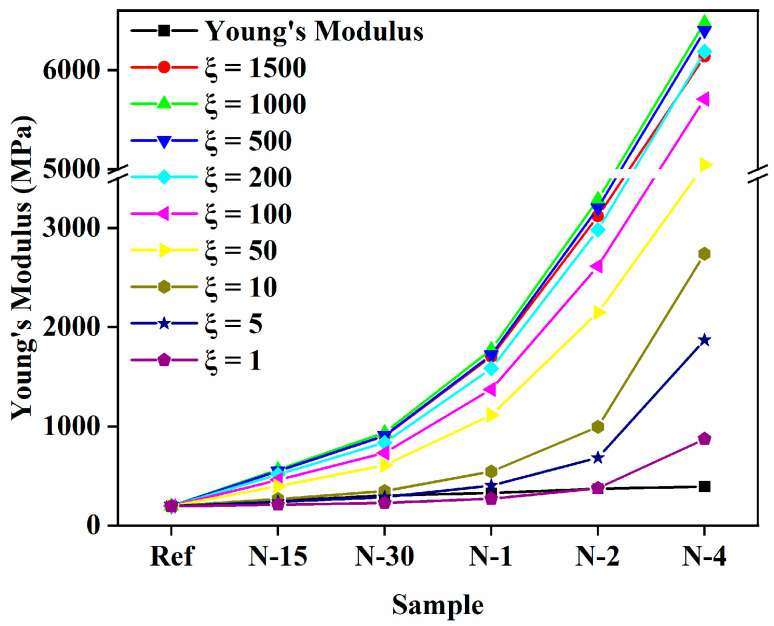
Effect of the exponential shape factor *ξ* on *E_c_* of the nylon 6 nanofiber-reinforced PUA composites.

For the Halpin–Tsai equation, shape factor *ξ* and orientation factor α were introduced, and modified the model as shown in Equations (12) and (13). In this research, *ξ* values were assumed and the graph was plotted for nanofiber-reinforced composites, as shown in Figure 4. However, this model usually unpredicted the *E_c_* of nanofiber-reinforced nanocomposites, and it was clearly found that the calculations were above the experimental data as volume fractions of the nanofibers increased in the composites. There was no significant difference observed for *ξ* > 200. Furthermore, the *ξ* (exponential shape factor) had the form of *ξ* = (2$l/d)e^{-av_{f}-b}$.

In this study, the *E_m_* of the PUA matrix and nylon 6 nanofibers were 196 MPa and 30 GPa, respectively. The orientation factor *α* was appropriated as 0.333. The effects of the aggregation/agglomeration coefficients *a* and *b* on the *E_c_* of the nanofiber-reinforced composites were estimated using the modified Halpin–Tsai equation. As depicted in Figure 4, no substantial changes were observed in the *E_c_* of the composites, and because of that, the values of aggregation/agglomeration coefficients predicted less than 200 to fit with the experimental *E_c_* of the composites.

**Figure 5:**
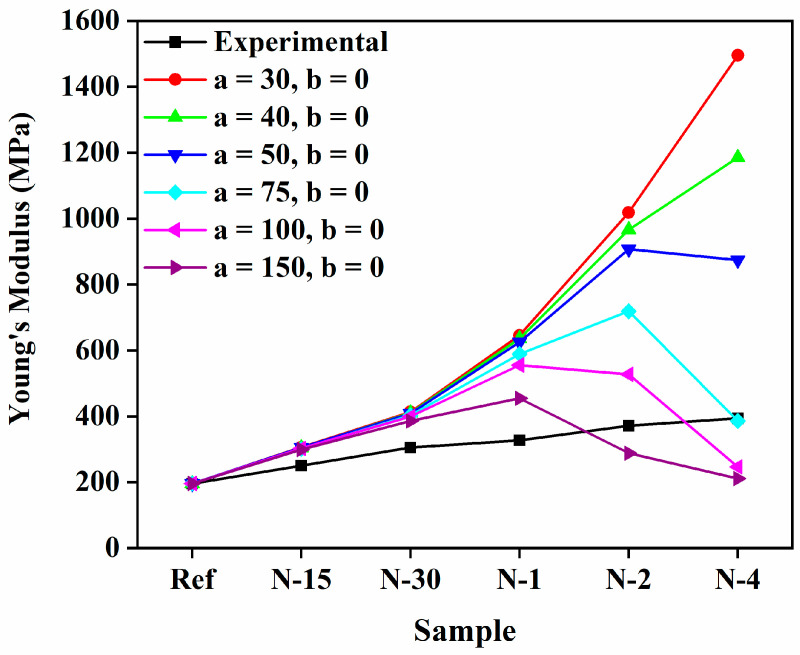
Effect of the aggregation/agglomeration coefficients *a* on *E_c_* of the nylon 6 nanofiber-reinforced PUA composites.

The effect of aggregation-related coefficient *a* nurtured to relent the fitted curves of *E_c_* of the nanofiber-reinforced composites at a high % of nanofibers, which demonstrated more aggregation established with multiplying nanofiber content. The aggregation-related coefficient *b* on the model curve for *E_c_* of the nanofiber-reinforced composites is depicted in Figure 5. The *E_c_* of composites was further adapted to tend lower for higher values of *b* at a high-volume fraction of nylon 6 nanofibers.

After periodically modifying the coefficients of aggregation *a* and *b*, complete adequacy to the experimentally achieved *E_c_* of the nylon 6 nanofiber-reinforced PUA composites was found when *a* = 30 and *b* = 3, and it could be modeled by Equations (12) and (13) with the following exponential shape factor:

(21) $$\xi =(2l/d)e^{-30v_{f}-3}$$

**Figure 6:**
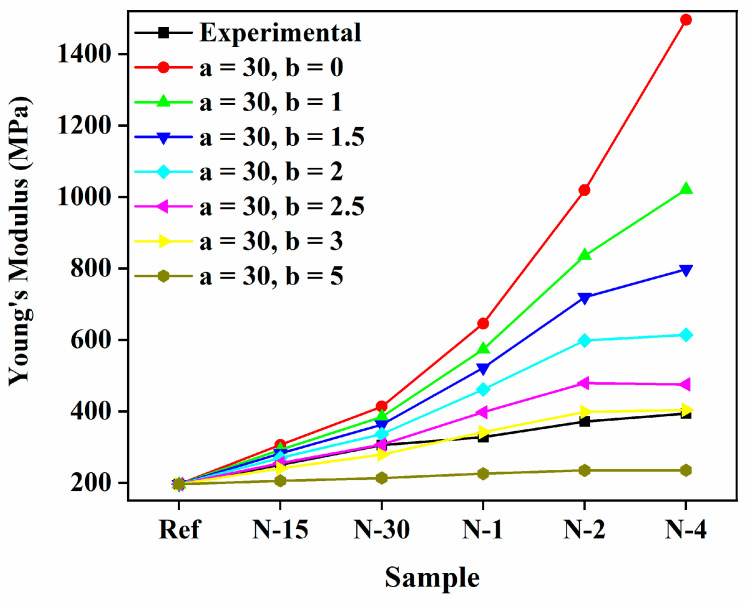
Effect of the aggregation-related coefficient *b* on *E_c_* of the nylon 6 nanofiber-reinforced PUA composites.

However, model Equations (12) and (13), associated with Equation (21), are termed the modified Halpin–Tsai equation for nanofiber-reinforced composites. Figure 6 depicts the comparison of experimental data with theoretical values of the nanocomposites adapted by the modified Halpin–Tsai equation model with exponential shape factor *ξ*. In this study, the shape factor *ξ* was picked to conceal the randomly aligned nanofibers with different volume fractions, and it underrated the value of *E_c_* of the nanofibers loading to a little below 0.008% volume fraction. Moreover, the mechanical characteristics of the composites at marginally higher wt% of nanofibers were a little overestimated using this modified model by reasonably extrapolating results.

**Figure 7:**
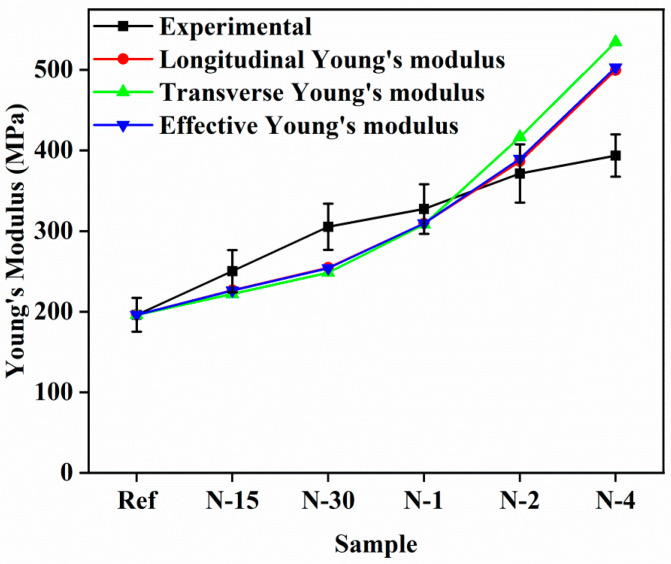
Comparison between the theoretical modeling results and experimental results for *E_c_* for the nanofiber-reinforced nanocomposites using the Hui–Shia model.

Hui–Shia model Equations (14) and (15) demonstrated higher aspect ratio estimation with the presence of randomly oriented fillers in matrix systems, and an increasing degree of fiber aggregation with increasing fiber content. For predicting the *E_c_* of the nanofiber-reinforced composites, *α’*, an inverse of the aspect ratio was correlated with the perfect interface geometric parameter *g* [31,40,41]. The inverse of the aspect ratio was smaller than 1 (*α’* = 0.001). Therefore, for calculating the geometric parameter *g*, Equation (19) was used, and it perfectly matched with the values from Equation (20). It is well known that the orientation of the dispersed phase has a substantial effect on the composite *E_c_*. It was evidenced from their geometry that disk- or plate-like fillers can contribute equal reinforcement in two directions if proportionately oriented, while fibers contribute primary reinforcement in one direction. In this case, the nanofibers were randomly oriented, and if the longitudinal modulus and transverse modulus were known, the effective modulus of the composite in all orthogonal directions [31] could be derived by the following equation:

(22) $$E_{c}=xE_{11}+\left(1-x\right)E_{22}$$

where *E_c_*_,_ *E*_11_, and *E*_22_ are effective, longitudinal, and transverse Young’s moduli of the composite, respectively. *x* is the constant to govern the stress transfer amongst the fibers and matrix systems in the composites, which is based on the curve fitting with experimental data (0 < *x* < 1). As shown in Figure 7, from Equations (14) and (15), longitudinal and inverse modulus predictions were obtained. The curve fitting with experimental results showed that factor *x* was calculated to be about 0.9, but it could only provide a rough estimation for *E_c_* of randomly oriented nanofiber-reinforced composites. The percentage errors of *E_c_* prediction for nanofiber-reinforced composites became much less than the experimental result data, and theoretical predictions appeared to overestimate experimental result data at higher volume fractions. The overestimated results could be based on the geometric constants engaged in the equation.

From the above analysis, we can identify that in order to obtain more precise predictions of the tensile properties of nanofiber-reinforced polymer nanocomposites, nanofiber aspect ratio, distribution of nanofibers, and volume fraction of the nanofibers are fundamentally important. These models simultaneously review these parameters and ensure a simple approach to predict the mechanical characteristics of the nanofiber-reinforced polymer nanocomposites. However, it is a significant challenge to establish a more comprehensive model capable of considering the nanofiber geometry, agglomeration, critical nanofiber length, and so on, which is the subject of future research.

## 5. Conclusions

A comparative study among the experimental results and several theoretical models of filler reinforcement was implemented to predict the mechanical characteristics of electrospun nylon 6 nanofiber-reinforced PUA nanocomposites. Our findings suggested that the theoretical results predicted employing the Pukanszky model and Nicolais–Narkis model are in close agreement with the tensile strength experimental values of the nanofiber-reinforced nanocomposites. Meanwhile, the Neilson and Halpin–Tsai models of tensile strength showed higher bound values than the experimental values for the high-volume fraction of the nanofibers in composites (for 0.033 and 0.066 *V_f_*). Additionally, theoretical values predicted using the modified Halpin–Tsai model showed close agreement with the available experimental values of Young’s modulus, due to the introduction of exponential shape factor *ξ* in the form of *ξ* = (2$l/d)e^{-av_{f}-b}$. Young’s modulus values evaluated using the Hui–Shia model and Halpin –Tsai were in the least agreement with the experimental values. The theoretical prediction applicable for a polymer nanocomposite with randomly dispersed high-aspect-ratio nanofibers has its advantages, since polymer nanofiber-reinforced nanocomposites are extensively used in human motion detection, self-healing electronic skins, sensors, fuel cells, epidermal electronics, microfluidic devices, and so on. It would be advantageous to estimate the mechanical properties of these composites using these modified models, before designing the final experiment.

## Figures and Tables

**Table 1:** Physical characteristics of the nylon 6 nanofiber-reinforced PUA nanocomposites.

Samples	Theoretical Density	Practical Density	wt% of Nanofibers	Volume Fraction of the Nanofibers	Volume Fraction of the PUA Matrix
PUA	1.19	1.1968	0	0	100
N-15	1.2	1.1964	0.0038	0.004	99.96
N-30	1.2	1.1957	0.0075	0.008	99.92
N-60	1.19	1.1944	0.015	0.017	99.83
N-2 h	1.19	1.1928	0.03	0.033	99.67
N-4 h	1.19	1.1918	0.06	0.066	99.34

**Table 2:** Mechanical properties of the N6/PUA nanocomposite films (modified from ref. [16]. copyright 2021 Wiley-VCH).

Sample		Tensile Strength at Break (MPa)	% Elongation at Break	Tensile Strength at Yield (MPa)	Young’s Modulus (MPa)
PUA films		15.20 ± 2.36	75.45 ± 7.74	5.78 ± 0.93	196.09 ± 20.99
N6/PUA nanocomposite films	N-15	17.42 ± 1.01	90.66 ± 3.12	6.71 ± 0.44	250.25 ± 26.33
	N-30	18.46 ± 0.82	86.22 ± 2.68	6.88 ± 1.07	305.34 ± 28.73
	N-60	20.88 ± 0.46	84.77 ± 5.31	7.45 ± 0.95	327.47 ± 30.82
	N-2 h	22.89 ± 0.50	89.44 ± 6	10 ± 2.31	371. 35 ± 36.11
	N-4 h	28.14 ± 2.36	86.55 ± 2.29	9.93 ± 1.28	393.72 ± 26.20

## Data Availability

Not applicable.
